# Immunosuppressive Effects of A-Type Procyanidin Oligomers from *Cinnamomum tamala*

**DOI:** 10.1155/2014/365258

**Published:** 2014-10-30

**Authors:** Liang Chen, Yang Yang, Pulong Yuan, Yifu Yang, Kaixian Chen, Qi Jia, Yiming Li

**Affiliations:** School of Pharmacy, Shanghai University of Traditional Chinese Medicine (SHUTCM), Shanghai 201203, China

## Abstract

Cinnamon barks extracts have been reported to regulate immune function; however, the component(s) in cinnamon barks responsible for this effect is/are not yet clear. The aim of this study is to find out the possible component(s) that can be used as therapeutic agents for immune-related diseases from cinnamon bark. In this study, the immunosuppressive effects of fraction (named CT-F) and five procyanidin oligomers compounds, cinnamtannin B1, cinnamtannin D1 (CTD-1), parameritannin A1, procyanidin B2, and procyanidin C1, from *Cinnamomum tamala* or *Cinnamomum cassia* bark were examined on splenocytes proliferation model induced by ConA or LPS. Then, the effects of activated compound CTD-1 on cytokine production and 2,4-dinitrofluorobenzene (DNFB) induced delayed-type hypersensitivity (DTH) response were detected to evaluate the immunosuppressive activity of CTD-1. It was found that CT-F and CTD-1 significantly inhibited the splenocyte proliferation induced by ConA or LPS. CTD-1 dose-dependently reduced the level of IFN-*γ* and IL-2 and intensively suppressed DNFB-induced DTH responses. These findings suggest that the immunosuppressive activities of cinnamon bark are in part due to procyanidin oligomers. CTD-1 may be a potential therapeutic agent for immune-related diseases.

## 1. Introduction 

Cinnamonis well known as a spice that is mainly distributed in Asia, South America, and the Caribbean [1].* Cinnamomum cassia*, the most popular type of cinnamon, has been used for thousands of years in traditional Chinese medicine (TCM) for treating various disorders such as chronic gastric symptoms, circulation disturbances, arthritis, and common cold. Other species, such as* C. japonica, C. burmannii,* and* C. chingii, *are used as cinnamon in some parts of China. The use of cinnamon barks to treat rheumatoid arthritis (RA), a chronic inflammatory autoimmune disease, was described in an ancient Chinese medical book “Tai Ping Sheng Hui Fang,” owing to its function of warming yang for dispelling cold. Many modern clinical experiences have also reported that cinnamon bark powder (or the prescriptions which contain cinnamon) is useful in treating RA although the key component(s) of this action is/are still unknown [2–4].

Cinnamon bark is reported as a good antiarthritic agent because of its anti-inflammation, pain relieving, and immune regulation function [5]. The anti-inflammatory activity of cinnamon species has been attributed to a series of procyanidins oligomers found in its barks [6]. Recently, literatures report that the water-soluble cinnamon bark extract had potential effects in regulating immune function* in vitro* and in preventing and treating inflammation-related diseases [7–9]. These results suggested that the water-soluble components in cinnamon, such as procyanidin oligomers, may play an important role in the therapeutic action of RA; however, the fact needs more studies to confirm.

Procyanidins oligomers are widely distributed in plants. They are mainly classified as A-type and B-type based on the linkage between the successive flavan-3-ol units. Previous studies found that cinnamon species contained both the A-type and B-type procyanidin oligomers [10, 11]. Recently, our studies found that* C. cassia* contained mainly B-type procyanidin dioligomer and trioligomer, whereas* C. tamala, *mainly used in Yunnan Province of China, contains mainly A-type procyanidin trioligomers. The different types of procyanidin oligomers may have different effects just as they have diverse antidiabetes mechanisms [12, 13].

To determine whether procyanidin oligomers in cinnamon species have immunosuppressive effects and whether the different types have varying effects on immunosuppression, five procyanidin oligomers isolated from cinnamon barks including three A-type procyanidin oligomers which are main constituents in* C. tamala* bark and two B-type procyanidin oligomers which are main constituents in* C. cassia* bark were evaluated for inhibitory effects of ConA-induced splenocyte proliferation. Furthermore, the anti-RA activity* in vitro* and* in vivo* of the most active compound CTD-1 among the five procyanidin oligomers was studied.

## 2. Materials and Methods 

### 2.1. Materials and Reagents

*C. cassia* and* C. tamala *were collected from Guangxi and Yunnan Provinces, respectively. The samples were botanically authenticated by Professor Guanyun Gu at the School of Pharmacy, Fudan University. The voucher specimens, numbers RG006 and RG007, were deposited at the Herbarium of the Department of TCM Chemistry, School of Pharmacy of Shanghai University of Traditional Chinese Medicine (Shanghai, China).

RPMI 1640 powder was purchased from Gibco. ConA and LPS were purchased from Sigma. [3H]-Thymidine (1 mCi/mL) was purchased from the Shanghai Institute of Atomic Energy. ELISA kits for IL-2 and IFN-*γ* were purchased from BD PharMingen (San Diego, CA, USA). 2,4-Dinitrofluorobenzene (DNFB) and other reagents were of analytical grade.

### 2.2. Experimental Animals

Female BALB/c mice, aged 6 weeks to 8 weeks, were purchased from the Shanghai Laboratory Animal Center, Chinese Academy of Sciences. The animals were housed in a specific pathogen-free environment (22 ± 1°C, RH 55 ± 5%, and 12 h light-to-dark cycle) at least 6 days before use. All procedures were carried out in accordance with the Chinese legislation on the use and care of laboratory animals and were approved by the respective university committees for animal experiments (Permission number: 2013018).

### 2.3. Extraction and Isolation

The preparation of procyanidin oligomers enriched extracts from two cinnamon species,* C. tamala* and* C. cassia *bark, was described in our previous paper [13]. Then the compounds were systematically isolated by using column chromatography and HPLC semipreparation. Briefly, 25 g of EtOAc fraction of* C. tamala* was dissolved in a small amount of MeOH, diluted with water, chromatographed on an open macroporous resin DM130 column (6.0 cm × 50.0 cm), and eluted with gradient of MeOH-H_2_O (0%, 10%, 40%, and 70% of MeOH) to yield 40% MeOH fraction 12.6 g, which was labeled CT-F. CT-F was dissolved in 10% aqueous acetonitrile, subjected to ODS-A-HG C18 column (4.0 cm × 35.0 cm), and eluted with 10% acetonitrile to yield six fractions (A1 to A6). A4 and A5 fractions were applied to Toyopearl HW-40F column chromatography (3.0 cm × 40.0 cm, eluted with 70% MeOH) to yield freeze-dried powder compounds** 1 **(300 mg) and** 2 **(230 mg), respectively. The A2 fraction was separately applied to preparative HPLC (12% acetonitrile) to produce freeze-dried powder compound** 3** (15 mg).

The* C. cassia* bark was processed by performing the same steps as those used for CT-F fraction to yield 40% MeOH fraction 2.7 g. Then, the fraction was subjected to Sephadex-LH20 column (3.5 cm × 50.0 cm, isocratically eluted with 50% MeOH) and, thereafter, ODS-A-HG C18 column chromatography (2.5 cm × 30.0 cm, isocratically eluted with 10% acetonitrile) to yield freeze-dried powder compounds** 4** (100 mg) and** 5** (40 mg).

### 2.4. Characterization of A-Type Procyanidin Oligomers (TAPOs) from CT-F

The obtained fraction (CT-F) and compounds (**1**–**5**) were characterized by high performance liquid chromatography (HPLC). HPLC analyses were performed on Waters ACQUITY UPLC H-class instrument equipped with a sample manager-FTN, quaternary solvent manager, column heater, photodiode array (PDA) detector, and Empower-Station for data collection and manipulation. An Agilent Extent-C18 column (5 *μ*m; 4.6 × 250 mm) was used in this experiment. The samples were analyzed using a solvent system with 0.1% acetic acid (A) and acetonitrile (B) under the following conditions: linear gradients from 12% to 18% B for 20 min, 45% solvent B for 25 min, 70% solvent B for 30 min, and 90% B for 35 min at a flow rate of 1 mL/min at 280 nm.

### 2.5. Cell Viability

Cell viability was assessed by performing MTT assay [14]. Briefly, splenocytes (4 × 10^5^ cells/well) were cultured in triplicate in the absence or presence of CT-F or five procyanidin oligomers in a 96-well plate for 48 h at 37°C in a 5% CO_2_ atmosphere for 48 h. The incubation mixture was washed out prior to addition of MTT reagent. Then, the cell was incubated with MTT (0.5 mg/mL) for 4 h. DMSO was then added to dissolve the precipitate after washing out the MTT, and O.D. values were read at 570 nm in a microplate reader.

### 2.6. Proliferation Assay

Splenic lymphocytes (4  ×  10^5^ cells/well) and CT-F or five procyanidin oligomers compounds in 96-well plates were cultured in triplicate for 48 h by using ConA (2 *μ*g/mL) or LPS (10 *μ*g/mL). Cells were pulsed at 0.25 *μ*Ci/well of [^3^H]-thymidine for 8 h before the end of the culture and then harvested onto glass fiber filters. Incorporated radioactivity was then measured by using a beta scintillation counter (MicroBeta Trilux, PerkinElmer Life Sciences, Boston, MA, USA).

### 2.7. Cytokine Measurement and DNFB-Induced DTH Reactions of CTD-1

Since the results of compounds activity screening showed that CTD-1 had the best immunosuppression effect on ConA-induced splenocyte proliferation, only CTD-1 was used for the cytokine and DNFB assay out of 5 compounds. Generally, splenocyte cells (4  ×  10^5^ cells/well) were incubated with 2 *μ*g/mL ConA and CTD-1 in 24-well plates. After 48 h, culture supernatants were harvested and stored at −20°C. IL-2, IFN-*γ* concentrations in supernatant were assessed by performing ELISA according to the manufacturer's instructions.

DNFB-induced DTH reaction of CTD-1 was examined according to a previously described method [15]. BALB/c mice were initially sensitized with 0.5% DNFB dissolved in acetone-olive oil (4 : 1) on each hind foot on days 0 and 1. On day 9, mice were challenged with 0.4% DNFB on both sides of their left ear. Vehicle, CTD-1 (3.3, 10, and 20 mg/kg), was administered to each group p.o. after the first day of initial sensitization. Five female BALB/c mice were prepared for each group. Ear swelling indicated the difference between the weights or thickness of the left and right ear patches, which were obtained from 8 mm punches 24 h after challenge.

### 2.8. Statistical Analysis

Results were expressed as mean ± SD, one-way analysis of variance (ANOVA) followed by Dunnett's test analysis was performed, and a value of *P* < 0.05 was considered significant.

## 3. Results

### 3.1. Structural Elucidation of Procyanidin Oligomer Compounds **1** to **5** (Figure 1)

**Figure 1:**
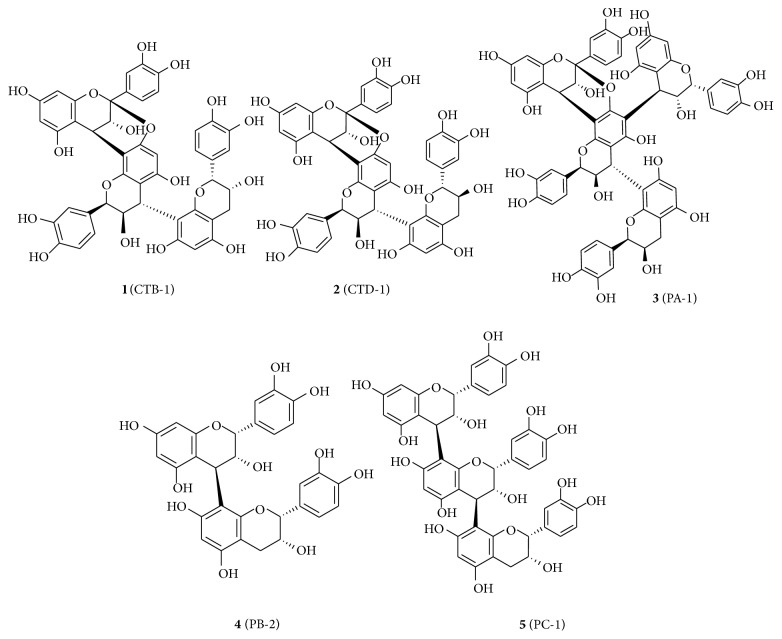
Chemical structures of compounds** 1** to** 5**.

Separation of CT-F by ODS and Toyopearl HW-40 columns led to the isolation of three A-type procyanidin oligomers (compounds** 1**–**3**), whereas separation of CC-F using the same columns led to the isolation of two B-type procyanidin oligomers (compounds** 4** and** 5**). The structure of procyanidin oligomer compounds** 1** to** 5 **was shown in Figure 1. The purity of each compound was detected more than 95% by using HPLC method (Supplementary Material available online at http://dx.doi.org/10.1155/2014/365258).

Compound** 1** was obtained as an off-white freeze-dried powder: [*α*]_*D*_^23^ + 65° (*c* = 0.1, MeOH). The ESI-MS spectra of** 1** showed the molecular ion [M-H]^−^ at* m/z* 863, which indicates an A-type interflavanoid linkage procyanidin trimer. ^13^C NMR (CD_3_OD, 100 MHz) spectrum of** 1** exhibited ketal resonance at *δ*_C_ 100.0, and the ^1^H NMR (CD_3_OD, 400 MHz) spectrum yielded AB system protons at *δ*_H_ 3.31 (1H, d, 3.4 Hz) and 4.17 (1H, d, 3.4 Hz), which indicates the presence of an A-type double-linked structure [16]. Carbon signals at *δ*_C_ 78.9 and 80.3 were consistent with C-2 resonances of epicatechin moieties. ^1^H and ^13^C NMR spectroscopic data (Supplementary Material) were compared with those in previous studies and revealed that** 1** was epicatechin-(4*β*→8, 2-O-7)-epicatechin-(4*β*→8)-epicatechin (cinnamtannin B1, CTB-1) [17, 18].

Compound** 2 **was obtained as an off-white freeze-dried powder: [*α*]_*D*_^23^ + 108° (*c* = 0.1, MeOH). The ESI-MS spectra of** 2** also showed the molecular ion [M-H]^−^ at* m/z* 863, which indicates an A-type procyanidin trimer. The NMR data of** 2** were similar to those of** 1**, except that aliphatic proton resonances appeared at *δ*_C_ 83.3, 70.1, and 30.7 and *δ*_H_ 3.94 (1H, d, 9.1 Hz), 3.67 (m), 3.04 (1H, dd, 16.2 Hz, 6.2 Hz), and 2.41 (1H, dd, 16.2 Hz, 10.1 Hz), which are consistent with the terminal unit as a catechin moiety. ^1^H and ^13^C NMR spectroscopic data (Supplementary Material) were compared with those in previous studies and revealed that** 2** was epicatechin-(4*β*→8, 2-O-7)-epicatechin-(4*β*→8)-catechin (cinnamtannin D1, CTD-1) [19, 20].

Compound** 3** was obtained as an off-white freeze-dried powder: [*α*]_*D*_^23^ + 46° (*c* = 0.1, MeOH). The ESI-MS spectra of** 3** exhibited a molecular ion [M-H]^−^ at* m/z* 1151, which suggests an A-type procyanidin tetramer. The ^1^H NMR (CD_3_OD, 400 MHz) spectrum of** 3 **showed the presence of two sets of meta-coupled protons at *δ*_H_ 6.00 (1H, d, 2.0 Hz) and 6.08 (1H, d, 2.0 Hz) and *δ*_H_ 5.95 (1H, d, 2.0 Hz) and 5.90 (1H, d, 2.0 Hz) along with a single proton at *δ*_H_ 6.10 (1H, s), which indicates C-4 →C-8′ (C-ring, D-ring) and C-4′′→C-6′ (I-ring, D-ring) linkages. In the ^13^C-NMR spectrum, the presence of three carbon signals at *δ*_C_ 78.7, 80.2, and 76.6 due to the C-2 of each flavan-3-ol unit suggests that the other flavan-3-ol units were epicatechin moieties. Compound** 3** was identified as epicatechin-(4*β*→8, 2-O-7)-[epicatechin-(4*β*→6)]-epicatechin-(4*β*→8)-epicatechin (parameritannin A1, PA-1) based on a direct comparison between its spectral data (Supplementary Material) and those of previous studies [21].

Compound** 4** was obtained as light brown freeze-dried powders: [*α*]_*D*_^23^ + 26° (*c* = 0.1, MeOH). ESI-MS spectra showed the molecular ion [M-H] at* m/z* 577, which suggested that** 4 **was a B-type procyanidin dimer. Carbon signals at *δ*_C_77.1, 79.7 (CD_3_OD, 100 MHz) were consistent with the C-2 resonances of epicatechin moieties [22]. The spectral data of** 4** (Supplementary Material) were consistent with epicatechin-(4*β*→8)-epicatechin (procyanidin B2, PB-2), which was also recorded in literature [23, 24].

Compound** 5** was obtained as a light brown freeze-dried powder: [*α*]_*D*_^23^ + 82° (*c* = 0.1, MeOH). ESI-MS spectra showed a single molecular ion [M-H]^−^ at* m/z* 865, which indicates that it was a B-type procyanidin trimer. Carbon signals at *δ*_C_ 77.1, 77.1, and 79.7 were consistent with C-2 resonances of epicatechin moieties. ^1^H and ^13^C NMR spectroscopic data (Supplementary Material) were compared with those in previous studies and revealed that** 5** was epicatechin-(4*β*→8)-epicatechin(4*β*→8)-epicatechin (procyanidin C1, PC-1) [24, 25].

### 3.2. Characterization of TAPOs from CT-F

**Figure 2:**
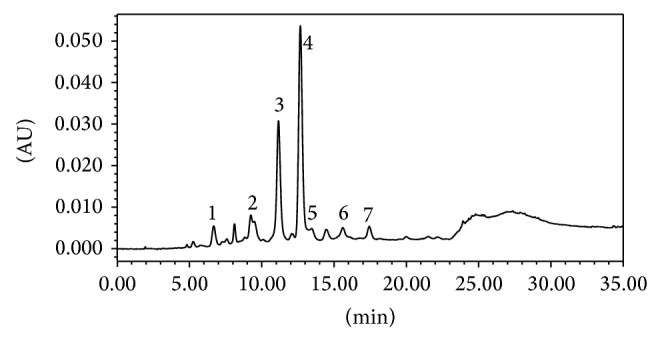
RP-HPLC chromatographic profile of procyanidin oligomers of CT-F, detected at 280 nm. 1: PA-1, 2: PB-2, 3: CTD-1, 4: CTB-1, 5: PC-1, and 6-7: A-type procyanidin trimers.

As shown in Figure 2, the retention time values of the main chromatographic peaks in CT-F were at 6.7, 9.2, 11.2, 12.7, 13.4, 15.6, and 17.4 min. These peaks were marked as 1 to 7. In comparison to the reference compounds isolated from two cinnamon barks, the peaks were identified as follows: 1 was PA-1; 2 was PB-2; 3 was CTD-1; 4 was CTB-1; 5 was PC-1. Peaks 6 and 7 were identified as A-type procyanidin trimers according to ESI-MS data that showed the presence of molecular ions at* m/z* 863 in the negative ion mode (Supplementary Material). Hence, CT-F may primarily contain A-type procyanidin because the main peaks in CT-F were CTD-1 and CTB-1, which are A-type procyanidin trimers.

### 3.3. CT-F Cytotoxicity on Splenocytes and Inhibition of ConA-Induced Splenocyte Proliferation

**Figure 3:**
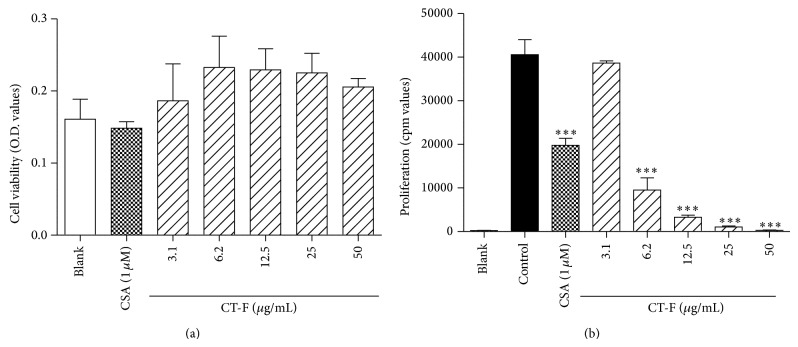
Cytotoxicity on splenocytes and inhibition on ConA-induced splenocyte proliferation of CT-F. (a) Cytotoxicity of CT-F on BALB/c mice splenocytes. The cells were incubated with different concentration of CT-F for 48 h. And the cell viability was measured by MTT assay. (b) Inhibition of CT-F on ConA-induced splenocyte proliferation. BALB/c mice splenocytes (4 × 10^5^ cells/well) were stimulated by ConA (2 *μ*g/mL) for 48 h in the presence of CT-F. Cells were then pulsed with 0.25 *μ*Ci [^3^H]thymidine 8 h before the end of the experiment and were assessed for [^3^H]thymidine incorporation. Effect of CSA (1 *μ*M) was set as positive group. Results are mean ± S.D. ^***^ *P* < 0.001, treatment group versus control.

The immunosuppressive effects of CT-F were evaluated* in vitro* by ConA-induced splenocyte proliferation. The results showed that CT-F has no cytotoxic effect on splenocytes at concentrations of 3.1 *μ*g/mL to 50 *μ*g/mL (Figure 3(a)).  Figure 3(b) revealed that CT-F had significant inhibition on splenocyte proliferation under ConA induction in a concentration-dependent manner. The IC_50_ value of CT-F on inhibiting lymphocyte proliferation was 5.5 *μ*g/mL.

### 3.4. Evaluating Immunosuppressive Effects of Compounds **1** to **5**

**Figure 4:**
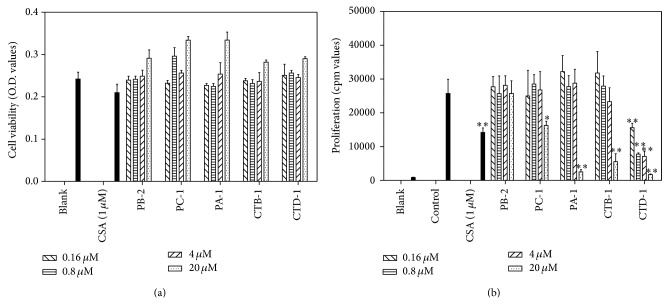
Cytotoxicity on splenocytes and inhibition on ConA-induced splenocyte proliferation of five procyanidin compounds** 1**–**5**. (a) Cytotoxicity of procyanidin compounds on BALB/c mice splenocytes. The cells were incubated with different concentration of procyanidin compounds for 48 h. And the cell viability was measured by MTT assay. (b) Inhibition of procyanidin compounds on ConA-induced splenocyte proliferation. BALB/c mice splenocytes (4 × 10^5^ cells/well) were stimulated by ConA (2 *μ*g/mL) for 48 h in the presence of different procyanidin compounds. Cells were then pulsed with 0.25 *μ*Ci [^3^H]thymidine 8 h before the end of the experiment and were assessed for [^3^H]thymidine incorporation. Effect of CSA (1 *μ*M) was set as positive group. Results are mean ± S.D. ^*^ *P* < 0.05, ^**^ *P* < 0.01, treatment group versus control.

The immunosuppressive effects of the five procyanidins were determined using the same method (Figure 4). It was found that all five test compounds have no cytotoxic effect at concentrations from 0.16 *μ*M to 20 *μ*M (Figure 4(a)).  Figure 4(b) showed that, even at a low concentration of 0.16 *μ*M, CTD-1 can inhibit ConA-induced splenocyte proliferation by 40%, suggesting that CTD-1 produced the best effects of all compounds. Other A-type procyanidin oligomers CTB-1 and PA-1 significantly suppressed ConA-induced splenocyte proliferation at 20 *μ*M. However, B-type procyanidin dimer PB-2 had no inhibitory effect even at 20 *μ*M. Meanwhile, B-type procyanidin trimer PC-1 had weakly effect on ConA-induced splenocyte proliferation, only reducing it by 37% at a high concentration of 20 *μ*M.

### 3.5. CTD-1 Inhibition of Mitogen-Induced Splenocyte Proliferation

**Figure 5:**
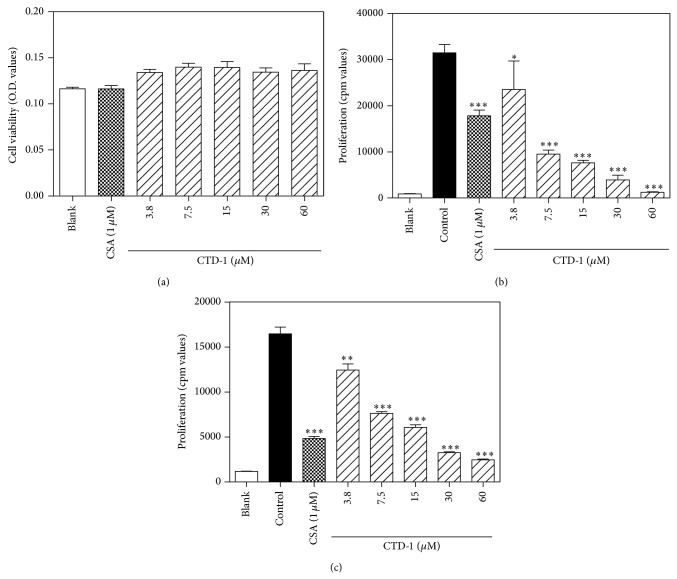
Cytotoxicity on splenocytes and inhibition on ConA or LPS induced splenocyte proliferation of CTD-1. (a) Cytotoxicity of CTD-1 on BALB/c mice splenocytes. The cells were incubated with different concentration of CTD-1 for 48 h. And the cell viability was measured by MTT assay. ((b) and (c)) Inhibition of CTD-1 on splenocyte proliferation induced by ConA (b) or LPS (c). BALB/c mice splenocytes (4 × 10^5^ cells/well) were stimulated by ConA (2 *μ*g/mL) or LPS (10 *μ*g/mL) for 48 h in the presence of CTD-1. Cells were then pulsed with 0.25 *μ*Ci [^3^H]thymidine 8 h before the end of the experiment and were assessed for [^3^H]thymidine incorporation. Effect of CSA (1 *μ*M) was set as positive group. Results are mean ± S.D. ^*^ *P* < 0.05, ^**^ *P* < 0.01, and ^***^ *P* < 0.001, treatment group versus control.

In order to further evaluate the immunosuppressive effect of CTD-1, two* in vitro* models, ConA and LPS induced splenocyte proliferation, were used in this experiment. The proliferation was suppressed in the CTD-1 treated splenocyte under ConA or LPS induction in a concentration-dependent manner (Figures 5(b) and 5(c)). The values of 4.38 ± 0.12 *μ*M and 6.62 ± 1.04 *μ*M correspond to two models induced by ConA and LPS. So the different of two values is reasonable.

### 3.6. Effects on Cytokine Production and DNFB-Induced DTH Response of CTD-1

**Figure 6:**
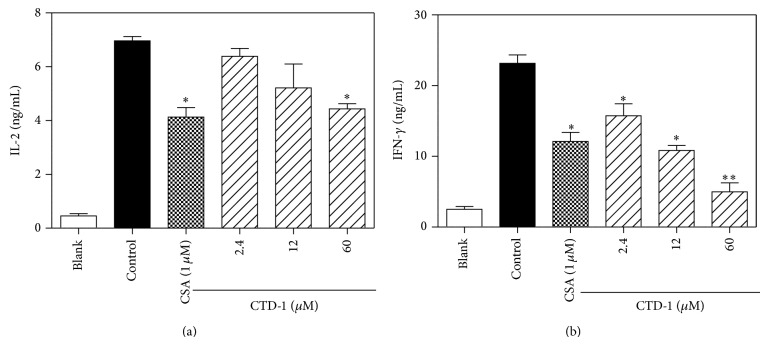
Effects of CTD-1 on cytokine production by ConA-stimulated splenocytes. Splenocytes (4 × 10^5^ cells/well) were incubated for 48 h with CTD-1 and ConA (2 *μ*g/mL). Culture supernatants were collected and analyzed by using ELISA for levels of IL-2 (a), IFN-*γ* (b). Effect of CSA (1 *μ*M) was set as positive group. Results are expressed as mean ± S.D. ^*^ *P* < 0.05, ^**^ *P* < 0.01 compared with control.

**Figure 7:**
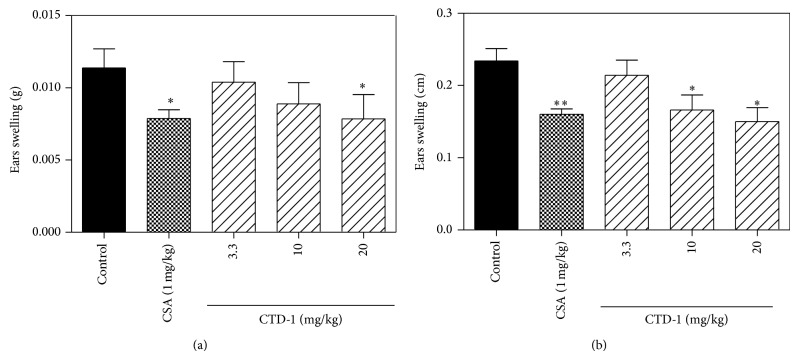
Effects of CTD-1 on DTH responses in BALB/c mice. BALB/c mice were initially sensitized with DNFB on days 0 and 1 and then challenged with DNFB on day 9. Vehicle and CTD-1 were administered after the first challenge. Ear swelling was calculated as the difference between the weights (a) or thickness (b) of left (DNFB treated) and right (untreated) ear punches 24 h after challenge. Treatment by CSA (1 mg/Kg) was set as positive group. Data are expressed as mean ± S.D. ^*^ *P* < 0.05, ^**^ *P* < 0.01, *n* = 5 mice/group.

The effects of CTD-1 on the production of Th1-type cytokines IL-2 and IFN-*γ* and effect on DTH responses in BALB/c mice were examined. As shown in Figure 6, IL-2 and IFN-*γ* productions were significantly decreased when the splenocyte cultures were exposed to CTD-1. In* in vivo* experiment, in DNFB-induced DTH, CTD-1 significantly suppressed ear weight (thickness) by 8.9% (7.6%), 29.4% (10.7%), and 36.2% (29.2%) at the dosage of 3.3, 10, and 20 *μ*M, respectively. These results suggested that CTD-1 might protect hosts from inflammatory reactions via strong inhibition of T cell functions (Figure 7).

## 4. Discussion

Since procyanidin oligomers compounds are difficult to purify, the regulating immune functions of cinnamon were mainly focused on extracts but not purified compounds in the previous studies [7, 8]. In this paper, the main procyanidin oligomers in cinnamon were successfully purified with the Toyopearl HW-40 and ODS-A-HG C18 gel chromatographic column. The HW-40 gel shows good ability in separation of the procyanidin oligomers with different degree of polymerization, whereas ODS C18 gel is good at separating the oligomers with different polarity and is also available for the isolation of isomers such as CTB-1 and CTD-1. The combined application of these chromatographic supports provides a good method for the isolation and purification of procyanidin oligomers, resulting in sufficient quantities of purified single procyanidin oligomers for active assay.

Our findings indicate that A-type procyanidin oligomers have better immunosuppressive effects than B-type procyanidin oligomers. Similar A-type procyanidin trimers, CTD-1 and CTB-1, showed significantly different effects. The linkage pattern between procyanidin units and the spatial configuration of hydroxyl group on C-ring of terminal unit might play an important role in immunosuppression.

Moreover, CTD-1 exhibits significant immunosuppressive effects* in vitro* and* in vivo*, which include suppression of lymphocyte proliferations induced by ConA and LPS, inhibition of Th1-type cytokines IL-2 and IFN-*γ* production, and inhibition of DTH response. These would result in the decrement of cellular and humoral immune responses. The immunosuppression appears to be mediated by T cells, indicating that the immune regulation effect of CTD-1 might inhibit T cell functions. Furthermore, CTD-1 possessed relative high immunosuppressive activities with low cytotoxicity.

Cytokines play an important role in the immune system and in immunomodulatory potential targets [26, 27]. IFN-*γ* plays a pivotal role in immune inflammatory reactions. IFN-*γ* can activate macrophages, which subsequently synthesizes TNF-*α* and proinflammatory chemokines to maintain inflammation [14]. CTD-1 inhibited IFN-*γ*, IL-2 productions, which suggested its possible therapeutic role in treating diseases that are associated with synthesis/release of these cytokines. To assess the immunosuppressive effects of CTD-1 on cellular and humoral immunity, a DTH model was used* in vivo*. The inhibitory effect of CTD-1 on T cell functions was confirmed in a murine DTH model. Ear swelling is recognized as the primary result of functions of antigen-specific* in vivo* CD4+ T cell response in DTH [28, 29]. Administration of CTD-1 strongly inhibited DNFB-induced ear swelling, which indicates its suppressive effect on T-cell-dependent immune responses. Thus, CTD-1 may protect hosts from inflammatory reactions via strong inhibition of T cell functions.

## 5. Conclusions

CT-F and CTD-1 exhibit low toxicity and have significant immunosuppressive effects, which can be attributed to their inhibition of T cell functions. In the meantime, our results indicate that the treatment of RA using cinnamon may be due in part to the procyanidin oligomers especially A-type. CTD-1 is a good candidate as a potential therapeutic agent for inflammatory and autoimmune diseases.

## Supplementary Material

The Supplementary Material contains detailed NMR spectrum data and HPLC chromatogram of five procyanidin oligomers and HPLC-MS chromatogram of CT-F extraction.

## Abbreviations

ConA: Concanavalin A
LPS: Lipopolysaccharide
DNFB: 2,4-Dinitrofluorobenzene
DTH: Delayed-type hypersensitivity
RA: Rheumatoid arthritis
TCM: Traditional Chinese medicine
TAPOs: A-type procyanidin oligomers
PB-2: Procyanidin B2
CTB-1: Cinnamtannin B1
CTD-1: Cinnamtannin D1
PA-1: Parameritannin A1
PC-1: Procyanidin C1.
