# Supplementary material for: Immunosuppressive Effects of A-Type Procyanidin Oligomers from Cinnamomum tamala
Source: Evid Based Complement Alternat Med. 2014 Oct 30;2014:365258. doi: 10.1155/2014/365258 (PMC4230008; doi:10.1155/2014/365258)
Supplement: Supplementary file 1 — The Supplementary Material contains detailed NMR spectrum data and HPLC chromatogram of five procyanidin oligomers and HPLC-MS chromatogram of CT-F extraction. [file 365258.f1.pdf]

Supporting information

Spectral data:

Table 1. The ^{1}H NMR (400MHz, CD_3OD) and ^{13}C NMR data of **1**

CTB-1	Upper unit		Middle unit		Terminal unit	
No	δ_c	δ_H	δ_c	δ_H	δ_c	δ_H
2	100		78.9	5.73 (br. s)	80.3	4.41 (br. s)
3	67.2	3.31 (d, 3.5 Hz)	72.6	4.15 (br. s)	67.5	3.89 (br. s)
4	28.8	4.17 (d, 3.4 Hz)	38.3	4.58 (br. s)	29.8	2.79-2.94 (m)
5	156.7		155.8		155.8	
6	98.3	5.99 (d, 2.2 Hz)	96.1	5.83 (s)	96.5	6.13 (s)
7	157.8		151.1		156	
8	96.5	6.04 (d, 2.2 Hz)	106.4		108.8	
9	154.1		151.8		155.5	
10	104.9		106.7		99.9	
1'	131.8		132.4		133.1	
2'	116.1	7.05 (d, 1.7 Hz)	116.7	7.34 (d, 1.7 Hz)	116.7	6.87 (d, 1.7 Hz)
3'	145.9		145.5		146.6	
4'	145.3		145.7		146.3	
5'	115.7	6.83-6.88 (d)	116	6.83-6.88 (d)	115.7	6.83-6.88 (d)
6'	119.8	6.76-6.79 (d, 1.7 Hz)	121.3	7.21 (d, 1.7 Hz)	119.4	6.76-6.79 (d, 1.7 Hz)

Table 2, The ^{1}H NMR (400MHz, CD_3OD) and ^{13}C NMR data of **2**

CTD-1	upper unit		middle unit		terminal unit	
No	δ_C	δ_H	δ_C	δ_H	δ_C	δ_H
2	100.1		78.6	5.50 (br. s)	83.3	3.94 (d, 9.1 Hz)
3	67.2	3.45 (d, 3.5 Hz)	72.5	4.05 (br. s)	70.1	3.67 (m)
4	28.9	3.98 (d, 3.5 Hz)	39.7	4.52 (br. s)	30.7	3.04 (dd, 16.2, 6.2Hz)<br>2.41 (dd, 16.2, 10.1Hz)
5	157.8		156.7		155.6	
6	98.4	5.93 (d, 2.2 Hz)	96.1	5.83 (br. s)	96.5	6.10 (br. s)
7	157.8		151.1		155.8	
8	96.5	6.00 (d, 2.2 Hz)	106.3		108.8	

9	154.2		151.7		155.4	
10	105.0		106.6		101.8	
1'	132.4		131.5		132.7	
2'	115.8	7.08 (d, 2.0 Hz)	115.9	7.23 (d, 1.7 Hz)	115.7	6.75 (d, 2.0 Hz)
3'	145.8		145.9		146.0	
4'	146.7		146.3		145.5	
5'	116.5	6.84 (d, 8.3 Hz)	116.1	6.83 (d, 8.1 Hz)	116.2	6.76 (d, 8.0 Hz)
6'	120.1	6.95 (dd, 8.3, 2.0 Hz)	121.1	7.08 (dd, 8.1, 1.7 Hz)	120.0	6.66 (dd, 8.0, 2.0 Hz)

Table 3. The ^{1}H NMR (400MHz, CD_3OD) and ^{13}C NMR data of **3**

PA-1	upper unit		middle unit		terminal unit		extent unit	
No	δ_C	δ_H (m, J/Hz)	δ_C	δ_H (m, J/Hz)	δ_C	δ_H (m, J/Hz)	δ_C	δ_H (m, J/Hz)
2	100.2		78.7	5.70 (br. s)	80.2	4.10 (br. s)	76.6	4.77 (br. s)
3	66.8	3.30 (d, 3.8)	72.4	3.96 (m)	67.4	3.65 (m)	71.4	4.13 (br. s)
4	28.9	4.26 (d, 3.6)	38.4	4.45 (br. s)	29.7	2.76-2.87 (m)	37.7	4.40 (br. s)
5	156.7		154.3		156.1		157.9	
6	98.3	6.00 (d, 2.0)	107.8		96.7	6.10 (s)	96.2	5.95 (d, 2.0)
7	157.9		148.4		155.5		159.5	
8	96.6	6.08 (d, 2.0)	106.9		108.8		96.6	5.90 (d, 2.0)
9	154.3		150.3		155.6		159.3	
10	104.9		107.2		99.9		99.4	
1'	132.3		131.7		132.8		131.7	
2'	115.8	7.17 (d, 1.7)	116.8	7.33 (d, 1.7)	115.5	6.67 (d, 1.5)	116.8	7.13 (br. s)
3'	145.6		145.9		145.8		146.3	
4'	146.8		146.3		145.4		146.3	
5'	116.1	6.90-6.95	116.1	6.85 (d, 8.2)	115.9	6.74 (d, 8.0)	116	6.90-6.95
6'	119.9	6.90-6.95	121.4	7.23 (dd, 8.2,2.0)	119.3	6.30 (br. d, 8.2)	120.7	6.95 (dd, 8.7, 1.8)

procyanidin B-2 (**4**) :

^{1}H NMR (CD_3OD, 400 MHz): δ_H 2.78 - 2.82 [1H, br. d, J = 16.7 Hz, H-4α (F)], 2.91 - 2.94 [1H, br. d, J = 15.2 Hz, H-4β (F)]，3.90 [1H, br. s, H-3 (C)], 4.27 [1H, br. s, H-3 (F)]，4.62 [1H, br. s, H-4 (C)]，5.04 [1H, s, H-2 (C)]，5.89 - 6.15 [3H, H-6 (A), H-8

(A), H-6 (D)]，6.89 - 7.12 [6H, H-2 (B,E), H-5 (B,E), H-6 (B,E)]. ^{13}C NMR (CD_3OD, 100 MHz): C-ring δ_C 35.7 (C-4), 72.1 (C-3), 78.3 (C-2); F-ring δ_C 28.3 (C-4), 65.6 (C-3), 75.7 (C-2); A-ring δ_C 155.1 (C-5), 99.1 (C-6), 156.9 (C-7), 94.7 (C-8), 156.4 (C-9), 99.9 (C-10); D-ring δ_C 155.1 (C-5), 99.1 (C-6), 156.9 (C-7), 105.9 (C-8), 153.1 (C-9), 101.4 (C-10); B, E-rings δ_C 113.9 (×2), 114.5 (×2), 117.8 (×2), 130.7, 131.2, 144.2 (×2), 144.5 (×2).

procyanidin C-1 (**5**)

^{1}H-NMR (CD_3OD, 400MHz): δ_H 2.80-2.97 (2H, m), 4.02(2H, brs), 4.33(1H, brs) , 4.72(2H, brs), 4.99(1H, brs), 5.07 (1H, brs), 5.22(1H, brs), 5.93-6.00 (4H, m), 6.70-7.32 (9H, m)

^{13}C-NMR(CD_3OD, 100MHz): δ_C 29.8, 37.3, 37.4, 66.8, 72.9, 73.4, 77.1 (×2), 79.7, 96.2, 96.6, 97.6, 100.6, 101.4, 107.2, 107.6, 115.1-116.1, 119.1-119.3, 132.1, 132.5, 132.7, 145.5-146.0, 156.6-158.2.

Figures

Fig.1 Common structure and sign of procyanidin oligomers.

Fig.2 RP-HPLC chromatographic profile of procyanidin oligomers compounds 1- 5 and CT-F, detected at 280nm. 1: PA-1, 2: PB-2, 3:CTD-1, 4: CTB-1, 5: PC-1, 6-7: procyanidin trimers. The HPLC purity of compounds **1**- 5 are more than 95%.

Fig. 3 Identification of the procyanidin oligomers in CT-F by LC-MS analysis.

The analyses were performed to identify the type of procyanidin oligomers in CT-F using a Shimadzu LC-20ADXR and a Thermo LCQ Fleet (Thermo Scientific) instrument. The outlet of the HPLC system was split (3:1) into the ESI-MS interface of the mass analyzer in the negative ion mode. ESI-MS analyses were performed under the following optimized conditions: source voltage, 4.5 kV; capillary voltage, –37 V; capillary temperature, 320 °C; sheath gas (N_2) flow; 40 arbitrary units (arb); and auxiliary gas (N_2) flow, 10 arb. Full-scan MS spectra (range, *m/z* 200 to 2000) were initially recorded during the chromatographic run.

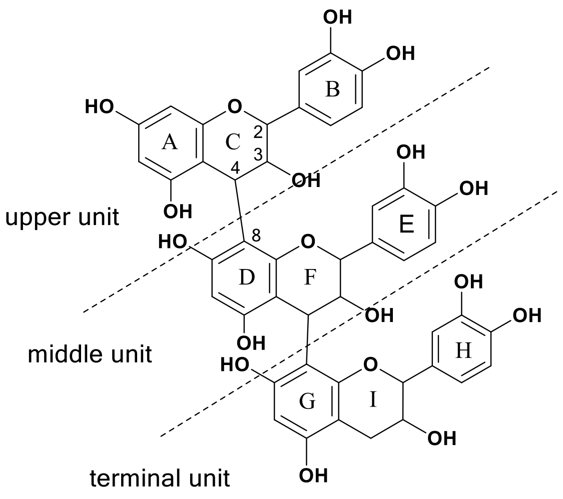
OH
OH
HO
B
A
C
2
3
4
OH
OH
upper unit
OH
OH
E
HO
8
D
F
OH
OH
middle unit
OH
OH
H
HO
G
I
OH
OH
terminal unit

Fig. 1

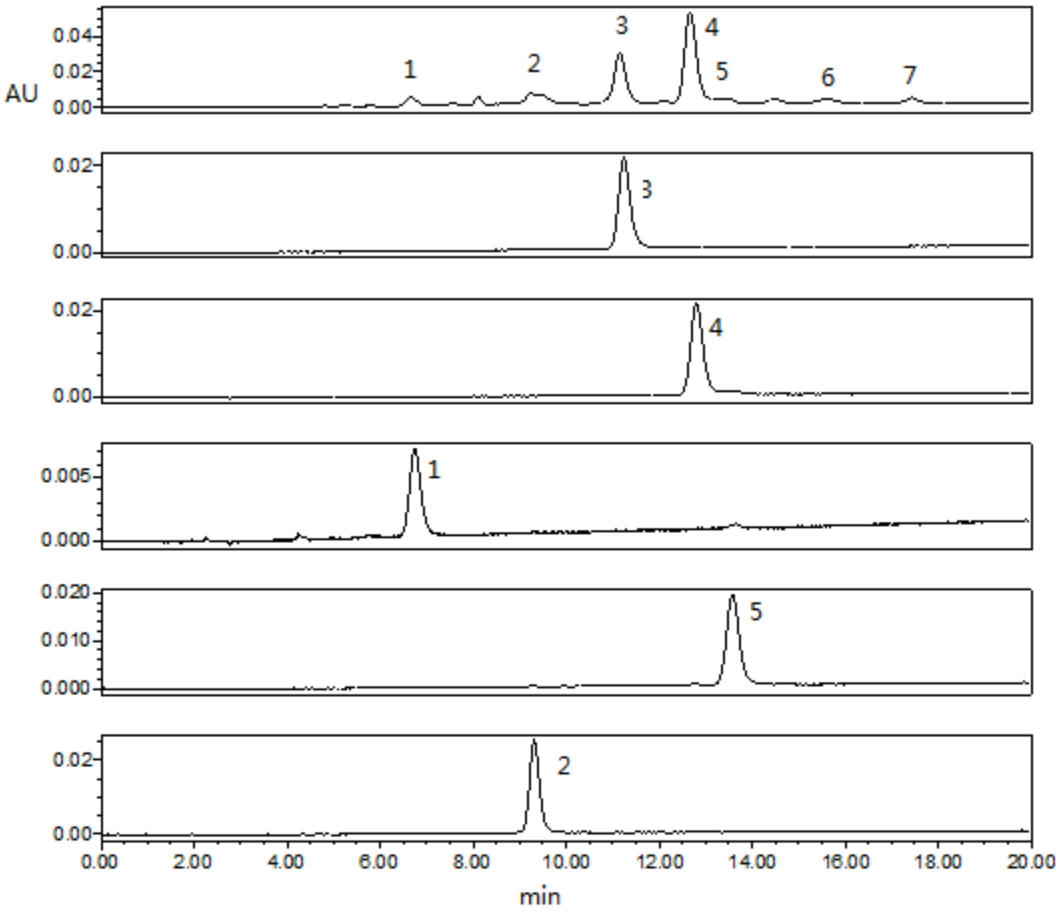
AU
0.04
0.02
0.00
1
2
3
4
5
6
7
0.02
0.00
3
0.02
0.00
4
0.005
0.000
1
0.020
0.010
0.000
5
0.02
0.00
2
0.00
2.00
4.00
6.00
8.00
10.00
12.00
14.00
16.00
18.00
20.00
min

Fig. 2

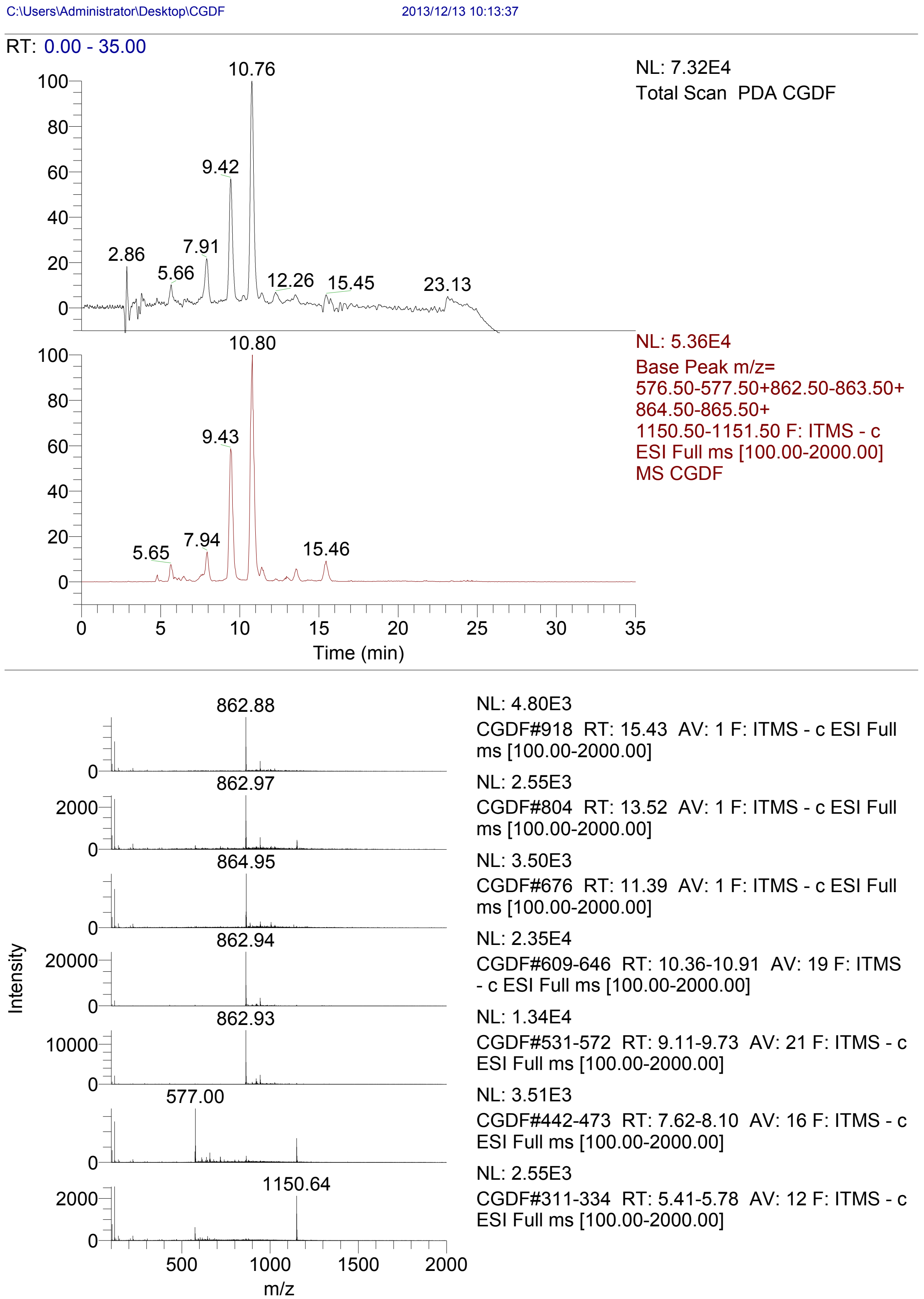

C:\Users\Administrator\Desktop\CGDF
2013/12/13 10:13:37
RT: 0.00 - 35.00
NL: 7.32E4
Total Scan PDA CGDF
10.76
9.42
7.91
2.86
5.66
12.26
15.45
23.13
NL: 5.36E4
Base Peak m/z=
576.50-577.50+862.50-863.50+
864.50-865.50+
1150.50-1151.50 F: ITMS - c
ESI Full ms [100.00-2000.00]
MS CGDF
10.80
9.43
7.94
5.65
15.46
Time (min)
862.88
NL: 4.80E3
CGDF#918 RT: 15.43 AV: 1 F: ITMS - c ESI Full ms [100.00-2000.00]
862.97
NL: 2.55E3
CGDF#804 RT: 13.52 AV: 1 F: ITMS - c ESI Full ms [100.00-2000.00]
864.95
NL: 3.50E3
CGDF#676 RT: 11.39 AV: 1 F: ITMS - c ESI Full ms [100.00-2000.00]
862.94
NL: 2.35E4
CGDF#609-646 RT: 10.36-10.91 AV: 19 F: ITMS - c ESI Full ms [100.00-2000.00]
862.93
NL: 1.34E4
CGDF#531-572 RT: 9.11-9.73 AV: 21 F: ITMS - c ESI Full ms [100.00-2000.00]
577.00
NL: 3.51E3
CGDF#442-473 RT: 7.62-8.10 AV: 16 F: ITMS - c ESI Full ms [100.00-2000.00]
1150.64
NL: 2.55E3
CGDF#311-334 RT: 5.41-5.78 AV: 12 F: ITMS - c ESI Full ms [100.00-2000.00]
Intensity
m/z

Fig. 3
